# Genome Sequence of *Bradyrhizobium tropiciagri* Strain CNPSo 1112^T^, Isolated from a Root Nodule of *Neonotonia wightii*

**DOI:** 10.1128/genomeA.01482-15

**Published:** 2015-12-17

**Authors:** Jakeline Renata Marçon Delamuta, Douglas Fabiano Gomes, Renan Augusto Ribeiro, Ligia Maria Oliveira Chueire, Renata Carolini Souza, Luiz Gonzaga Paula Almeida, Ana Tereza Ribeiro Vasconcelos, Mariangela Hungria

**Affiliations:** a Embrapa Soja, Soil Biotechnology, Londrina, Paraná, Brazil; b CAPES, SBN, Brasília, Federal District, Brazil; c CNPq, Brasília, Federal District, Brazil; d UFPR, Department of Microbiology, Curitiba, Paraná, Brazil; e LNCC, Labinfo, Petrópolis, Rio de Janeiro, Brazil

## Abstract

CNPSo 1112^T^ is a nitrogen-fixing symbiont of perennial soybean, a tropical legume forage. Its draft genome indicates a large genome with a circular chromosome and 9,554 coding sequences (CDSs). Operons of nodulation, nitrogen fixation, and uptake hydrogenase were present in the symbiotic island, and the genome encompasses several CDSs of stress tolerance.

## GENOME ANNOUNCEMENT

Biological nitrogen fixation performed by some prokaryotes that convert the atmospheric nitrogen (N_2_) into ammonia is considered the most important biological process after the photosynthesis (1, 2). The most effective contribution occurs with bacteria collectively known as rhizobia in symbiosis with leguminous plants (1, 2). The genus *Bradyrhizobium* is particularly important in the tropics, associated with a variety of legumes, including grain producers, trees, pastures, and green manure (3–6). The versatility of *Bradyrhizobium* goes beyond nitrogen fixation; for example, it can give important contribution to the degradation of agrochemicals (7). The genus encompasses strains adapted to a variety of ecosystems and living styles, from stem-nodulating bacteria with photosynthetic and nitrogen-fixing properties (8), to root-nodulating species showing high host specificity (9). Recently, the new species *Bradyrhizobium tropiciagri* was described (10), and here we report the draft genome of the type strain CNPSo 1112^T^ (=SMS 303^T^ = BR 1009^T^ = SEMIA 6148^T^ = LMG 28867^T^), isolated from a root nodule of perennial soybean [*Neonotonia wightii* (Wight & Arn.) Lackey], a forage from Africa that grows well in several tropical countries, including Brazil.

To access the bacterial genome sequence, total DNA was extracted using the DNeasy blood and tissue kit (Qiagen) and processed at the Ion PGM platform (Life Technologies) at the LNCC, Petrópolis, Brazil. The FASTQ files were *de novo* assembled by Newbler version 2.9 (Roche). Shotgun sequencing allowed coverage of approximately 68-fold, and the genome analysis revealed that CNPSo 1112^T^ has one circular chromosome. Sequences were submitted to RAST (11), and the genome was estimated at 9,767,314 bp, assembled in 189 contigs. Annotation identified 9,554 coding sequences (CDSs). The analysis at the SEED system (11) allowed the classification of 40% of the CDSs in 504 subsystems. Interestingly, CNPSo 1112^T^ had a higher number of putative genes in the category amino acids and derivatives (15.6%) than in the carbohydrates (14.5%).

The draft genome of CNPSo 1112^T^ confirms that it belongs to the *Bradyrhizobium elkanii* superclade (10). We investigated the genes of the symbiotic island, and verified that the strain carries the nodulation operon with two copies of the regulatory *nodD* gene, followed by *nodABCSUIJ_nolO_nodZ*. The *nodZ* gene has been suggested to play an important role in determining host specificity, being present in many tropical rhizobia (12). In addition, there are the *nif* genes coding for the synthesis of the molybdenum-iron nitrogenase. The symbiotic island also carries genes coding for the hydrogenase uptake enzyme, which can confer higher efficiency to the nitrogen fixation process, recovering partially the energy lost with the obligatory evolution of H_2_ (13). Spread in the genome are also several CDSs related to stress tolerance, an important feature for survival in the tropics, including the categories of osmotic and oxidative stresses, cold and heat shock, detoxification, stress response, and periplasmic stress.

**Nucleotide sequence accession numbers**. This whole-genome shotgun project has been deposited at DDBJ/EMBL/GenBank under the following accession numbers: SUBID (SUB987619), BioProject (PRJNA287624), BioSample (SAMN03784761), Accession (LFLZ00000000). The version described in this paper is the first version.
